# Low Absolute Lymphocyte Count Associated With Anti‐Thymocyte Globulin Induction May Be a Predictor of Early Cytomegalovirus Infection in Pediatric Heart Transplantation

**DOI:** 10.1111/petr.70136

**Published:** 2025-07-13

**Authors:** Allyson Chan, Robert Tan, Sofia Tan, Micheal Kuhn, Natalie Shwaish, Richard Chinnock, Huyentran Tran, Erik Frandsen

**Affiliations:** ^1^ Loma Linda University Children's Hospital Loma Linda California USA

## Abstract

**Background:**

Cytomegalovirus (CMV) can cause serious morbidity in transplant patients. Lymphocyte depletion by anti‐thymocyte globulin (ATG) may persist for 6 months post‐induction, and lower absolute lymphocyte counts (ALC) may increase the risk for CMV. We sought to find the relationship between ALC at induction and CMV viremia in the first 6 months post‐transplant.

**Methods:**

An analysis of transplant recipients within a set period of time with uniformed induction therapy and CMV prophylaxis. ATG was a primary component of induction and ganciclovir or valganciclovir as prevention of CMV infection. The cohort was dichotomized into low ALC (≤ 0.3 × 10^9^ cells/L) and high ALC (> 0.3 × 10^9^ cells/L) based on lowest ALC during the first 14 days and their clinical characteristics analyzed. CMV viremia was defined as PCR value of > 137 IU/mL regardless of symptoms.

**Results:**

A total of 44 patients were included in this study. CMV viremia occurred in 18% (*n* = 8). Patients were more likely to develop CMV if post‐induction ALC was **≤** 0.3 × 10^9^ cells/L (28% vs. 0%, *p* = 0.029) and remained significant when adjusted for CMV serostatus (*p* = 0.04). The total ATG dose (7.5 vs. 4.5 mg/kg) was not predictive of CMV (37% vs. 17%, *p* = 0.3) nor was treatment for rejection (25% vs. 14%, *p* = 0.5. Rejection occurred in 7% compared to 20% of patients in the low vs. high ALC group (*p* = 0.4).

**Conclusion:**

In pediatric heart transplant recipients, there is a higher incidence of CMV if ALC ≤ 0.3 × 10^9^ cells/L during induction, regardless of serostatus. Low ALC levels during induction may identify a high‐risk group that could benefit from an altered CMV prophylactic regimen.

## Graphical abstract

In pediatric heart transplant recipients, there is a higher incidence of cytomegalovirus if ALC ≤ 0.3 × 10^9^ cells/L during induction, regardless of serostatus. Low ALC levels during induction may identify a high‐risk group that could benefit from an altered CMV prophylactic regimen.

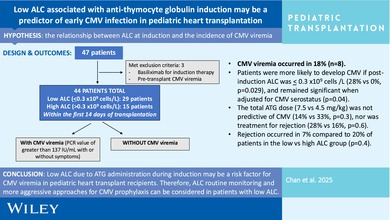

**Abbreviations**
ALC: absolute lymphocyte count
ATG: antithymocyte globulin
CMV: cytomegalovirus
EBV: Epstein Barr Virus
eGFR: estimated glomerular filtration rate
ICU: intensive care unit
PCR: polymerase chain reaction
PSI: proliferation signal inhibitor
WBC: white blood cell

## 1 Background

Cytomegalovirus (CMV) infection can lead to serious end‐organ dysfunction and graft loss in solid organ transplant patients. Among heart transplant recipients, CMV infection is associated with higher rates of intensive care unit (ICU) readmission and increased mortality [1]. CMV infection occurs in approximately 18% of adult and 21% of pediatric heart transplant patients [2, 3, 4].

Risk factors for CMV infection include induction and maintenance immunosuppression, donor/recipient CMV serostatus, leukopenia, concurrent EBV viremia, and treatment of allograft rejection [2]. In pediatric heart transplant patients, induction immunosuppression is most commonly anti‐thymocyte globulin (ATG) or basiliximab. Studies have not shown a significant difference in incidence of CMV infection between the two agents [5]. ATG is generally dosed to achieve CD3 counts < 25 cells/mm^3^; however, such levels may not always correlate with the absolute lymphocyte count (ALC) [6]. The lymphodepleting effects of ATG given during induction therapy have been demonstrated up to 6 months post‐transplantation. During this period of increased immunosuppression, the risk of CMV infection is highest and valganciclovir is recommended to prevent infection [7].

Several studies have described a correlation between low ALC and CMV viremia in adult solid organ transplantation [8, 9]. Pre‐transplant lymphopenia was associated with short‐term mortality and a higher incidence of post‐transplant infections in deceased donor liver transplant patients [10]. In adult heart transplant recipients receiving basiliximab induction, CMV infection within 60 days post‐transplant was associated with low ALC regardless of ganciclovir prophylaxis [11].

There is insufficient data addressing low ALC levels as a potential risk factor for early CMV infection in pediatric heart transplantation. Therefore, this study aims to describe the relationship between ALC at induction and the incidence of CMV viremia within the first 6 months post‐transplantation in pediatric heart transplant recipients.

## 2 Methods

We conducted a retrospective single‐center analysis of pediatric heart transplant recipients at Loma Linda University Children's Hospital between June 2018 and July 2024. During this time period, all patients received the same induction therapy and immunosuppression per protocol, resulting in a uniformity of therapy during the first 6 months after transplantation. Patients with pre‐transplant CMV viremia, previous transplantation, or who did not receive ATG during the induction period were excluded. The cohort was dichotomized into two groups based on the lowest ALC within the first 14 days of transplantation: low ALC (≤ 0.3 × 10^9^ cells/L) and high ALC (> 0.3 × 10^9^ cells/L). ALC was not drawn in consistent time intervals, but nadir was selected from the available ALC levels within 14 days post‐transplant. The lower limit of normal for ALC was defined as 0.3 × 10^9^ cells/L, consistent with other studies [12, 13]. From those two groups, we identified the primary outcome of CMV viremia, defined as a PCR value of > 137 IU/mL with or without symptoms.

**FIGURE 1:**
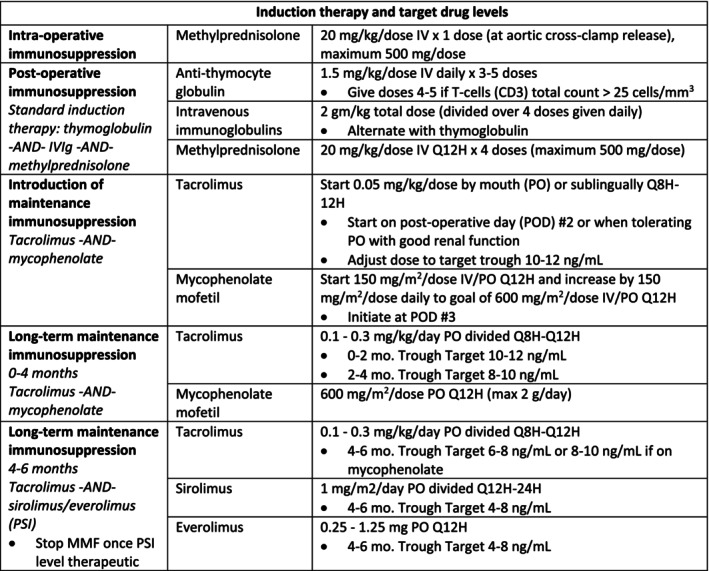
Induction therapy and target drug levels.

Our center's induction and maintenance immunosuppression protocol is outlined in Figure 1. ATG remained a principal therapy with a cumulative dose of 4.5 mg/kg. An additional 3 mg/kg was given if the CD3 count was > 25 cells/mm^3^ after receiving the initial doses. Patients received one dose intraoperatively and four doses of methylprednisolone immediately post‐transplant without a taper. No immunosuppression was given prior to transplantation. ALC values were obtained but did not guide ATG dosing.

All patients received CMV prophylaxis therapy for 3 months, including high‐risk CMV serostatus patients. CMV PCR was monitored as routine at monthly intervals for three occurrences, followed by 6, 9, and 12 months, then annually thereafter. Patients who were at lower risk of developing CMV (donor+/recipient+, donor−/recipient+, or donor−/recipient−) received oral valganciclovir 10 mg/kg/dose daily or twice daily for those who were high risk of EBV viremia (maximum 450 mg per dose). High‐risk individuals (donor+/recipient− serostatus) received intravenous ganciclovir 5 mg/kg/dose every 12 h (or adjusted for renal function) and transitioned to an oral valganciclovir dose of 7 * body surface area * estimated GFR twice daily (maximum 450 mg/dose) once oral intake was advanced.

Patients who developed CMV viremia received IV ganciclovir 5 mg/kg/dose every 12 h with or without CMV immune globulin for moderate or severe symptoms. Oral valganciclovir treatment dosing of 7 * body surface area * estimated GFR twice daily (maximum 900 mg/dose) was given to those who were asymptomatic or had mild symptoms. Treatment was continued until viral load was undetectable.

Institutional review board approval was obtained. A review of the patient's medical records included the following: patient's demographics, date of transplantation, CD3 count during the induction period, ALC within 14 days of transplant, and treated episodes of allograft rejection. In addition, CMV serostatus and duration of CMV prophylaxis, initial time of CMV viremia, immunosuppression at diagnosis, WBC, and evidence of concurrent EBV infection were recorded and analyzed.

Continuous variables are presented as median and interquartile range and compared by Mann–Whitney U testing. Categorical variables are presented as number and percentage and compared by Pearson Chi‐squared or Fisher's exact test as appropriate. Time to event data are compared by Kaplan–Meier analysis (IBM SPSS, Version 29, Armonk, NY).

## 3 Results

A total of 44 patients were included in the study. The median age at transplantation was 11.3 years (IQR 1.2, 15.3 years). All patients received antithymocyte globulin (ATG) as induction therapy. 80% received higher‐dose ATG (7.5 mg/kg) and 20% received lower‐dose ATG (4.5 mg/kg). At 6 months post‐transplant, all patients were on tacrolimus. The second immunosuppressive agent was mycophenolate mofetil in 61% and proliferation signal inhibitor (PSI) in 39%. No patient was on chronic corticosteroids. CMV donor/recipient serostatus was low risk (donor−/recipient−) in 10 (23%), medium risk (donor−/recipient+ or donor+/recipient+) in 22 (50%), and high risk (donor+/recipient−) in 12 (27%) (Table 1).

**TABLE 1:** Characteristics of low and high ALC groups.

Characteristic	Entire cohort (*n* = 44)	Low ALC (*n* = 29)	High ALC (*n* = 15)	*p* (high vs. low ALC)
Age at transplant, median (IQR), years	11.3 (1.2, 15.3)	11.6 (5.5, 15.3)	1.5 (0.5, 13.0)	0.25
Antithymocyte globulin induction total dose, no (%)				**0.02**.
7.5 mg/kg	35 (80%)	20 (69%)	15 (100%)	
4.5 mg/kg	9 (20%)	9 (31%)	0 (0%)	
Minimum ALC within 14 days of transplant, median (IQR), ×10^9^ cells/L	0.23 (0.15, 0.6)	0.18 (0.10, 0.22)	0.75 (0.60, 0.94)	**0.028**
CD3 at induction, median (IQR), cells/mm^3^	44 (31, 103)	37 (17, 62)	61 (41, 110)	0.8
Immunosuppression at 6 months, no. (%)				0.9
Mycophenolate and tacrolimus	27 (61%)	18 (62%)	9 (60%)	
PSI and tacrolimus	17 (39%)	11 (38%)	6 (40%)	
CMV serostatus, no. (%)				0.4
D−/R−	10 (23%)	5 (17%)	5 (33%)	
D−/R+ or D+/R+	22 (50%)	15 (52%)	7 (47%)	
D+/R− (CMV mismatch)	12 (27%)	9 (31%)	3 (20%)	
CMV high‐risk serostatus (D+/R−), no. (%)	12 (27%)	9 (31%)	3 (20%)	0.5
Valganciclovir prophylaxis dosing, no. (%)				0.6
10 mg/kg daily	31 (70%)	19 (66%)	12 (80%)	
10 mg/kg BID	4 (9%)	3 (10%)	1 (7%)	
7 mg*BSA*eGFR BID (max 900 mg daily)	9 (20%)	7 (24%)	2 (13%)	

*Note:* The bolded values in Table 1 are the statistically siginficant results.

CMV viremia occurred in 8 of 44 patients (18%) at a median time from transplant of 3.2 months (IQR 1.8, 3.6 months). Of those 8 patients, 4 were asymptomatic. Two patients had GI effects (diarrhea), and one had bronchiolitis with associated rhinovirus/enterovirus infection. At the time of CMV viremia, 5 of 8 patients (62.5%) were receiving valganciclovir prophylaxis. Three patients not on prophylaxis had already completed 3 months of prophylaxis after transplant. In this group, the median duration from discontinuation of valganciclovir to detection of CMV viremia was 22 days. Treatment in three patients consisted of oral valganciclovir. Five patients received IV ganciclovir, two of whom received additional CMV immune globulin. One individual required maribavir for recalcitrant CMV viremia and was ultimately referred for CMV cytotoxic T lymphocyte therapy.

Compared to patients with CMV viremia, those without had a higher median minimum ALC within 14 days of transplant (0.13 vs. 0.29, *p* = 0.028). ALC nadir occurred within the first 7 days in 39 patients (89%) and after a median of one dose of ATG. ALC continued to fluctuate above the nadir, even with subsequent dosages of ATG received. Rates of rejection, CD3 count at induction, high‐risk CMV donor/recipient serostatus (donor+/recipient−), ATG dose, and immunosuppression drug regimen at 6 months were similar between groups (Table 2). At the time of CMV viremia, the median WBC was 5.01 × 10^9^/L (IQR 3.31, 7.79), and 2 of 8 patients (25%) had concomitant EBV viremia.

**TABLE 2:** Characteristics of patients with and without CMV viremia.

Characteristic	CMV Viremia (*n* = 8)	No CMV Viremia (*n* = 36)	*p*
Minimum ALC within 14 days of transplant, median (IQR), ×10^10^ cells/L	0.13 (0.1, 0.25)	0.29 (0.18, 0.6)	**0.028**
Antithymocyte globulin induction total dose, no. (%)			0.3
7.5 mg/kg	5 (63%)	30 (83%)	
4.5 mg/kg	3 (37%)	6 (17%)	
Treatment of rejection, no. (%)	2 (25%)	5 (14%)	0.5
CD3 at induction, median (IQR), cells/mm^3^	48 (20, 91)	43 (33, 103)	0.8
Immunosuppression at 6 months, no. (%)			0.7
Mycophenolate and tacrolimus	4 (50%)	23 (64%)	
PSI and tacrolimus	4 (50%)	13 (36%)	
CMV high‐risk serostatus (D+/R−), no. (%)	4 (50%)	8 (22%)	0.1

*Note:* The bolded values in Table 2 are the statistically siginficant results.

**FIGURE 2:**
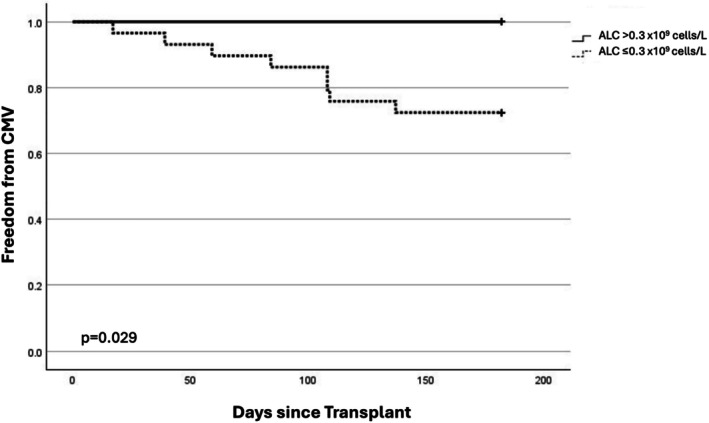
Kaplan–Meier curve depicting freedom from CMV viremia after transplant between the low and high ALC group.

Twenty‐nine patients were stratified into the low ALC group and fifteen into the high ALC group. CMV viremia was more commonly observed in the low ALC group compared to the high ALC group (28% vs. 0%, *p* = 0.029). The Kaplan–Meier curve illustrates freedom from CMV viremia between these two groups (Figure 2). Patients in the high ALC group were more likely to have received the higher total ATG dose compared to the low ALC group (100% vs. 69%, *p* = 0.02). The median minimum ALC in the low ALC group was 0.18 (IQR 0.1, 0.22) and the median minimum ALC in the high ALC group was 0.75 (IQR 0.6, 0.94) (*p* = 0.028). Other characteristics including patient age at transplant, high‐risk CMV donor/recipient serostatus (donor+/recipient−), and immunosuppression at 6 months were similar between groups (Table 1).

## 4 Discussion

In this retrospective, single‐center study, we examined the incidence of and identified risk factors for CMV viremia among a cohort of pediatric heart transplant recipients. CMV viremia occurred in 18% of our cohort at a median of 3.2 months from transplant. This incidence is comparable to other pediatric studies. A large study of 271 pediatric solid organ transplant recipients observed a similar incidence of CMV viremia (15%), with 21% of heart recipients developing CMV [4]. Furthermore, another large study of 393 pediatric solid organ transplant recipients demonstrated the incidence of CMV viremia in 11.2% of heart transplant recipients while on prophylactic therapy and 9.3% after the completion of prophylactic therapy [14].

In our study, patients who developed CMV viremia had a lower minimum absolute lymphocyte count (ALC) in the first 14 days post‐transplant. This finding is not unexpected. The adaptive immune response, through the action of T lymphocytes, is important for controlling viral infections. Solid organ transplant recipients, in whom T‐cell number and function are reduced, are at increased risk of viral infection. We routinely use ATG for induction therapy for all heart transplant recipients to deplete circulating lymphocytes. Whether additional ATG is given after the initial 4.5 mg/kg is dependent on CD3 count. In our cohort, the proportion of patients who received the higher total dose of ATG was greater in those without CMV viremia, which may reflect a more robust immune system, reducing the risk of CMV infection.

Interestingly, the ALC nadir often occurred after the first dose of ATG, and the ALC would trend up thereafter despite receiving additional dosages of ATG. ALC nadir should not be a determining factor in the total dose of ATG during induction, as it was seen to fluctuate regardless of the amount of ATG received. Having ALC ≤ 0.3 × 10^9^ cells/L at any point during the induction period may be an indicator for CMV viremia. Because all patients received induction therapy, we cannot comment on the risk of ATG to the development of CMV. However, other studies have demonstrated an increased risk of CMV when using induction therapy [15].

All patients received CMV prophylaxis with valganciclovir for 3 months after transplant, the dose dependent on the donor/recipient serostatus and EBV serostatus risk. However, we were not able to demonstrate an association between high‐risk donor/recipient serostatus and CMV viremia, possibly due to low numbers. Although a greater proportion of patients who developed CMV viremia were high‐risk compared to those without CMV viremia, this did not reach statistical significance (50% vs. 22%, *p* = 0.1). Deficiency in CMV‐specific immunity after transplant, whether functional due to immunosuppression or because of a lack of prior exposure to CMV, is the major risk factor for developing CMV disease [16]. Twenty‐seven percent of our cohort were high‐risk based on donor+/ recipient− serostatus. This compares favorably with a study of 271 pediatric solid organ transplant recipients, in whom 34% were high‐risk serostatus [14]. Patients who are < 18 months can have passively acquired maternal CMV IgG antibodies and may not have intrinsic CMV‐specific T‐cell immunity. Just one patient in our cohort who developed CMV viremia was < 18 months at transplant and was high‐risk serostatus.

The immunosuppression regimen was similar between patients with and without CMV viremia. Although all patients received induction therapy, which has been identified as a risk factor for CMV viremia, none received chronic corticosteroids. Use of PSI has been associated with lower risk of CMV infection in kidney transplant recipients and adult heart transplant recipients [17, 18].

Because graft rejection is treated with more aggressive immunosuppression, which may increase the risk of CMV infection, we evaluated the incidence of rejection. Although treated rejection was more common in patients with CMV viremia, it did not meet significance (25% vs. 14%, *p* = 0.5).

Our findings that CMV infection was isolated to those with ALC nadir of ≤ 0.3 × 10^9^ cells/L in the first 14 days post‐transplant may help guide the duration of post‐transplant CMV prophylaxis. We may consider extending the duration of prophylaxis beyond the typical 3 months or increasing the dose of prophylaxis in those with low ALC. In those who develop untoward side effects of valganciclovir past that time, the use of preemptive monitoring of CMV for at least 3–4 weeks after discontinuation may be considered. Careful monitoring is crucial since higher doses and durations of valganciclovir therapy may increase the risk of kidney dysfunction and bone marrow suppression [19]. Alternative therapies may include the concomitant use of CMV hyperimmunoglobulin in addition to longer duration valganciclovir. Some evidence suggests that the combination of CMV hyperimmunoglobulin and antiviral therapy may offer increased protection against CMV viremia and may be useful in patients with low ALC. However, the use of CMV hyperimmunoglobulin may have its own challenges, including infusion reactions and renal dysfunction [20, 21]. CMV cytotoxic T lymphocyte therapy may also be considered for patients with ganciclovir‐resistant CMV reactivation or in those who are unable to reconstitute adequate antiviral T‐cell immunity despite traditional therapies [22]. Letermovir may serve as a possible alternative in the future for our patient population [23].

There were several limitations to this study. First, it was single‐centered and retrospective in nature with only 44 patients, and there was an overall low number of CMV viremia cases. On the other hand, the baseline characteristics seem to be representative of the general pediatric heart transplant population. Second, only patients who underwent induction with ATG were included in this study, which is known to cause bone marrow suppression and a lasting effect for up to 6 months. Therefore, it may not be appropriate to extrapolate these results to patients receiving other induction therapies which do not include ATG. Third, this study focuses on the minimum ALC, instead of average, within 14 days of heart transplantation because ALC was not a routine monitoring parameter and the frequency of ALC levels was not standardized for all patients. It is also difficult to assess if patients received an adequate dose of valganciclovir since ganciclovir levels are not monitored; only estimated GFR‐based dosing was performed. Perhaps, utilizing ganciclovir therapeutic drug monitoring based on area under the curve could be beneficial to ensure adequate antiviral coverage, thereby reducing the percentage of patients developing CMV viremia while on prophylactic therapy [24, 25]. Breakthrough CMV infection despite the best prophylactic therapy can still happen. In this case, CMV genotyping may be useful to determine optimal treatment [26]. Fourth, age‐dependent lymphocyte counts were not considered when dichotomizing the patients into the high or low ALC groups. However, among the seven patients who were transplanted at < 6 months of age, only three were in the low ALC group, and one of those developed CMV viremia. This demonstrates that despite the evidence showing that lymphocyte counts are generally lower in infancy and increase by 6 months of age, a minimum ALC threshold may play a greater role in the development of CMV viremia [27]. Adjusting for age‐dependent lymphocyte counts may be beneficial in future studies with larger sample sizes.

In conclusion, low ALC due to ATG administration during induction may be a risk factor for CMV viremia in pediatric heart transplant recipients. Therefore, it may be valuable to routinely monitor ALC at induction and to consider more aggressive or alternative approaches for CMV prophylaxis in patients with low ALC. Although this study utilized a uniform induction and immunosuppression protocol and included patients with similar baseline characteristics, future large, multicentered studies are still warranted to determine the optimal dosing and duration of antiviral prophylactic therapy for pediatric heart transplant patients with low ALC.

## Disclosure

The authors have nothing to report.

## Data Availability

The data that support the findings of this study are available from the corresponding author upon reasonable request.
